# Polymer-Derived Ceramic Functionalized MoS_2_ Composite Paper as a Stable Lithium-Ion Battery Electrode

**DOI:** 10.1038/srep09792

**Published:** 2015-04-08

**Authors:** L. David, R. Bhandavat, U. Barrera, G. Singh

**Affiliations:** 1 Department of Mechanical and Nuclear Engineering, Kansas State University, Manhattan, KS 66506, USA

## Abstract

A facile process is demonstrated for the synthesis of layered SiCN-MoS_2_ structure via pyrolysis of polysilazane functionalized MoS_2_ flakes. The layered morphology and polymer to ceramic transformation on MoS_2_ surfaces was confirmed by use of electron microscopy and spectroscopic techniques. Tested as thick film electrode in a Li-ion battery half-cell, SiCN-MoS_2_ showed the classical three-stage reaction with improved cycling stability and capacity retention than neat MoS_2_. Contribution of conversion reaction of Li/MoS_2 _system on overall capacity was marginally affected by the presence of SiCN while Li-irreversibility arising from electrolyte decomposition was greatly suppressed. This is understood as one of the reasons for decreased first cycle loss and increased capacity retention. SiCN-MoS_2_ in the form of self-supporting paper electrode (at 6 mg·cm^−2^) exhibited even better performance, regaining initial charge capacity of approximately 530 mAh·g^−1^ when the current density returned to 100 mA·g^−1^ after continuous cycling at 2400 mA·g^−1^ (192 mAh·g^−1^). MoS_2_ cycled electrode showed mud-cracks and film delamination whereas SiCN-MoS_2_ electrodes were intact and covered with a uniform solid electrolyte interphase coating. Taken together, our results suggest that molecular level interfacing with precursor–derived SiCN is an effective strategy for suppressing the metal-sulfide/electrolyte degradation reaction at low discharge potentials.

Layered transition metal dichalcogenides (TMDs) such as molybdenum disulfide (MoS_2_) and tungsten disulfide (WS_2_) have garnered increased research interest because of applications in several emerging areas such as hydrogen storage^1^, chemical catalysis^2^, double-layer capacitors and rechargeable metal-ion battery electrodes^3^,^4^,^5^,^6^,^7^,^8^,^9^. Application as Li-ion battery electrode is particularly of interest because of weak van der Waals interactions between TMD layers and unique conversion chemistry with Li that allows continuous Li-ion cycling with lower volume expansion and pulverization compared to alloying anodes such as silicon and metal oxides.^10^,^11^ Theoretically, conversion reaction in one mole of MoS_2_ leads to four moles of stored Li+ ions resulting in a specific capacity of 670 mAh·g^−1^ (approximately 1.8 times the traditional graphite anode)^12^.

Reports on electrochemical performance of bulk MoS_2_ began in the 1980s when TMDs were investigated as cathode material for use in Li-metal batteries.^13^ However, safety issues lead to discontinuation of such batteries. Recent advances in nanotechnology, such as development of efficient liquid phase exfoliation methods,^14^,^15^,^16^,^17^,^18^,^19^,^20^ improved understanding of the electrode/electrolyte interfaces, and recent success with fabrication of single layer MoS_2_ transistors and membranes^21^,^22^ have motivated researchers to reconsider nanostructured MoS_2_ as potential Li host material. Highly porous MoS_2_ or MoS_2_/carbon electrodes prepared by hydrothermal and solvothermal techniques, gas-phase reaction of MoO_3_ with H_2_S or S vapor, and thermal decomposition of ammonium thiomolybdate have demonstrated performance improvements in response to challenges presented by MoS_2_ nanosheet use.^23^,^24^,^25^,^26^,^27^,^28^,^29^,^30^,^31^,^32^ As a result, capacity values as high as ~ 1200 mAh·g^−1^ have been achieved for such electrodes, particularly at low active weight loadings.^33^ More recently, MoS_2_-based hybrid nanocomposites (obtained by interfacing with carbon nanotubes or graphene) have been of interest because CNT or graphene offers high electrical conductivity^34^, thereby allowing high rate capability and reversibility.^35^,^36^,^37^,^38^,^39^,^40^,^41^,^42^ Nevertheless, some new challenges have emerged that hinder the introduction of TMD nanosheets for practical applications. These challenges include: (a) high costs due to complex procedures that limit production in large quantities (gram levels or higher), (b) capacity degradation for thick electrodes or the low volumetric capacity of nanostructured/porous electrode design, (c) low thermodynamic and chemical stability in moisture^21^ and degradation reaction with the battery electrolyte at low discharge potentials.^17^ Efforts to cost-effectively produce large quantities of TMDs flakes via chemical exfoliation are promising; therefore, a matter of intense research^19^,^20^.

Here, we report a one-step facile approach to synthesize a TMD/glass composite material. This approach could prove vital in retaining the useable (reversible) Li-ion capacity of TMD electrodes by mitigating the effect of phase-III electrolyte degradation reaction and polysulfide dissolution typically observed in these materials. The composite consists of a Si-based polymer-derived ceramic (or PDC)^43^,^44^,^45^,^46^ chemically interfaced with the surfaces of exfoliated MoS_2_. PDCs are high-temperature glasses prepared by thermal decomposition of organosilicon polymers. Monolayer thick films of PDC can be formed on a variety of substrate materials to achieve resistance to oxidation and chemical degradation without compromising the physical properties of base material; PDC/CNT and PDC/graphene are some examples.^43^,^44^,^45^,^46^,^47^,^48^ Liquid-phase polymeric precursor allowed easy dispersion and functionalization (attainment of molecular level interfacing) of exfoliated MoS_2_ flakes. Polymer molecules diffuse within exfoliated sheets,^49^ forming an alternating MoS_2_ and ceramic-layered morphology on pyrolysis, thereby exhibiting ideal candidate material for rechargeable battery electrodes. The composite has a tap density of ~ 1.5 g.cm^−3^ and could be processed as either a thick film on copper foil or freestanding paper with active weight loading as high as 6 mg.cm^−2^. This layered morphology may be essential for achieving long-term stable Li-cycling in Li-S and Li-ion batteries because it puts a check on the TMD/organic electrolyte degradation reaction observed at low discharge potentials.^17^,^50^,^51^

## Experimental

### Material preparation

**Figure 1:**
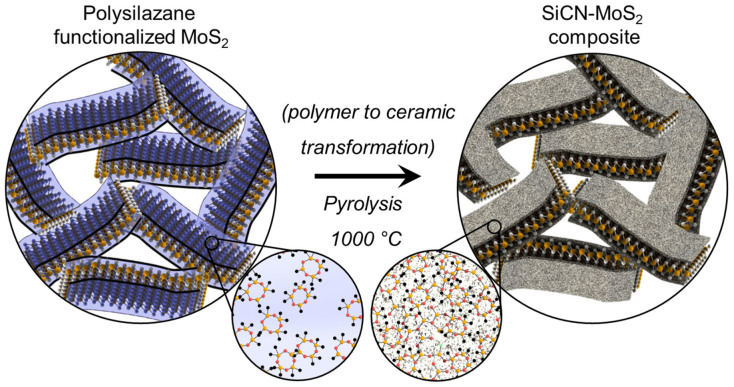
Schematic representation showing synthesis of SiCN-MoS_2_ composite from in-situ pyrolysis of polysilazane molecules. Liquid-phase polysilazane functionalized MoS_2_ flakes undergo organic-to-inorganic transformation at 1000°C in flowing nitrogen leading to SiCN-MoS_2_-SiCN type morphology. Sheet like morphology of MoS_2 _is retained.

Acid-treated MoS_2_ was prepared in a manner similar to our recent work on TMDs, described in Ref. 17 and 18. SiCN-MoS_2_ composite nanosheets were prepared following the procedures shown in Figure 1. Approximately 30 wt. % of poly(ureamethylvinyl)silazane (commercial name: Ceraset™, Clariant) was added to acid-treated MoS_2_, stirred for 24 h, and dried at 100°C in an inert atmosphere. To allow cross-linking of the intercalated polymer, the dried polymer-MoS_2_ mix was heated to 400°C in nitrogen (held at 15 min) and then heated at 1000°C for 1 h, resulting in thermal decomposition of the polymer into amorphous SiCN ceramic on MoS_2_ surfaces. Under these processing conditions, the polymeric precursor showed a ceramic yield of approximately 70 to 75 wt. % [see Supplementary Figure S1], resulting in approximately 20 wt. % of SiCN ceramic in SiCN-MoS_2_ composite.

### Cell assembly and testing

Traditional electrode: These were prepared using active materials (exfoliated MoS_2_ or SiCN-MoS_2_ composite), acetylene black (Alfa Aesar, 99.9%) as conducting agent and polyvinylidene fluoride (Sigma Aldrich) as a binding agent with a weight ratio of 8:1:1, respectively. Few drops of N-methyl pyrrolidone (Fisher) were added to obtain homogenously viscous slurry. Approximately 125 *μ*m uniformly thick coating was then prepared on copper current collector (9 *μ*m thick) and dried in an inert medium at 80°C for 4 h. They were then punched into circles of diameter 1.4 cm for use as working electrode in a coin cell (2032 coin cell) with Li-metal foil acting as the counter electrode. A polyethylene monolayer membrane (Celgard) soaked in 1.0 M of LiPF_6_ in EC: DMC electrolyte (Novolyte Technologies) separated the two electrodes. The cells were assembled in a high-precision argon glove box.

Paper-based electrode: Freestanding papers were prepared in a manner similar to recent work.^52^ Dispersion of SiCN-MoS_2_ composite with 20 wt. % graphene oxide^53^ was prepared in 1:1 water: Isopropanol solution. The dispersion was then vacuum-filtered through a 10 *µ*m filter membrane (HPLC grade, Millipore). The paper was separated from the membrane and thermally reduced at 500°C under argon atmosphere for 2 h. Because GO to rGO yield is approximately 50% [Supplementary Figure S2], the amount of rGO in the final paper was approximately 10 wt. % of the final composite. Four-point electrical conductivity of the paper was observed to be 0.11 S/cm. The heat-treated paper was then punched into small circles and directly utilized as a working electrode in the Li-ion battery half-cell.

### Material characterization

Scanning electron microscopy (SEM) was performed by use of Carl Zeiss EVO 10 SEM and transmission electron microscopy (TEM) was carried out on Philips CM 100 TEM (100 kV). EDX map was obtained using FEI Company Nova NanoSEM 430 with an Oxford X-Max Large Area Analytical EDS silicon drift detector (SDD) (80mm^2^). Material characterization was made using an X-ray diffractometer (XRD) operating at room temperature with nickel-filtered Cu K*α* radiation (*λ* = 1.5418 Å). Thermogravimetric analysis (TGA) was performed using Shimadzu 50 TGA (limited to 800°C). Samples weighing, ~ 2.5 mg, were heated in a platinum pan at a rate of 10°C/min in air flowing at 20 mL/min. Surface chemical composition of the powdered specimens were determined using X-ray photoelectron spectroscopy (XPS), by PHI Quantera SXM with Al K*α* monochromatic X-radiation (beam size < 9 *μ*m) at 45° angle of incidence. The assembled cells were tested using Arbin BT2000 multichannel potentiostat in atmospheric conditions. The batteries were cycled from 10 mV to 3V at a constant current density of 100 mA.g^−1^ during discharge and charge cycles.

## Results and discussion

**Figure 2:**
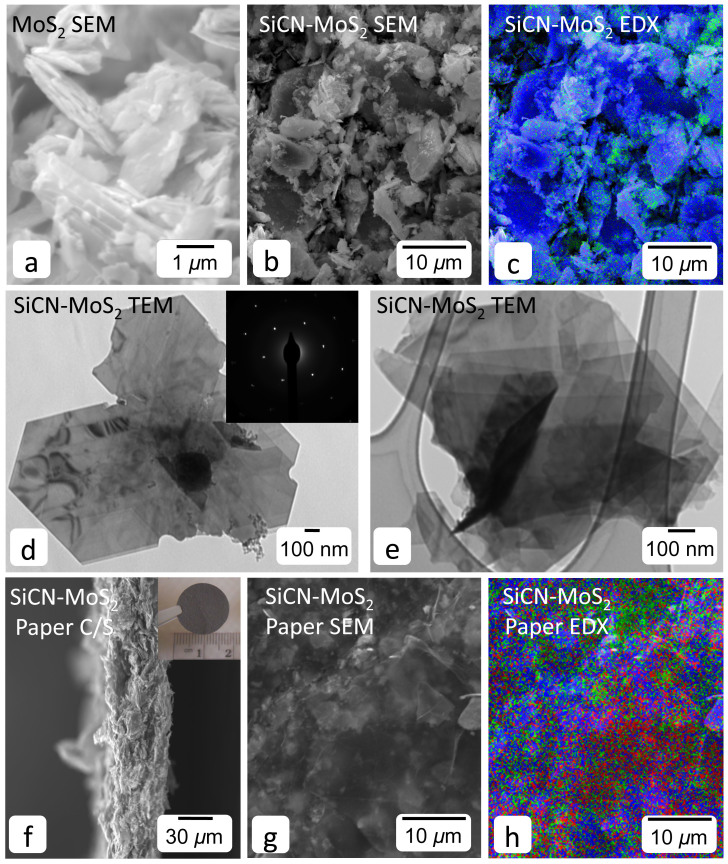
Material characterization. SEM micrograph of (a) acid-treated MoS_2_ show stacked sheets with lateral dimension of 2 *μ*m to 5 *μ*m. (b) SEM image of SiCN/MoS_2_ and (c) its corresponding EDX map show presence of Mo (blue), Si (Green) and C (red) distributed in the composite. (d,e) TEM images of SiCN/MoS_2_ sheet composite show intact morphology of MoS_2_ sheets after pyrolysis. The SAED pattern in the insert corresponds to crystalline MoS_2_. SEM images of SiCN/MoS_2_ freestanding paper (f) cross-section, (g) top view and (h) corresponding EDX map. The insert in Figure (f) is an optical photograph of SiCN/MoS_2_ freestanding and flexible paper.

Schematic representing synthesis of MoS_2_-SiCN composite is presented in Figure 1. Electron microscopy of the acid-treated MoS_2_ specimen demonstrated a large number of layered MoS_2 _sheets with lateral dimensions varying between 2 *μ*m to 5 *μ*m (Figure 2(a)). SiCN functionalized MoS_2_ composite also showed layered morphology, as shown in the SEM and TEM images (Figures 2(b–e)). The EDX map of SiCN-MoS_2_ in Figure 2(c) (Mo-blue, Si-green and C-red) shows an unevenly spread amorphous SiCN ceramic intercalated into MoS_2_ sheets. Interlayer separation and presence of pores or gaps was evident in SiCN-MoS_2_ composite. The SAED pattern in the insert of Figure 2(d) is similar to crystalline MoS_2_ pattern, indicating the presence of intact sheets even after pyrolysis. The cross-section, top view, and EDX map of freestanding papers prepared by vacuum filtration (approximately 10 wt. % rGO) are presented in Figures 2(f–h).

X-ray diffraction data for MoS_2_, polymer-derived SiCN, SiCN-MoS_2_ powder, and freestanding paper is presented in Supplementary Figure S3. XPS analysis was performed on the composite material to ascertain the presence and chemical functionalization of MoS_2_ by SiCN ceramic phase (Supplementary Figure S4). High-resolution elemental Mo3d XPS spectra showed doublets at 227.8 eV and 230.9 eV. The doublets are deconvoluted into the original Mo-S state at 226.8 eV and 227.8 eV due to the Mo-Si/Mo-C bond, and the high energy 230.9 eV peak could be due to Mo-O bonds with multiple oxidation states of Mo.^54^,^55^,^56^,^57^ A high-resolution sulfur peak emerged as a doublet at 160.7 eV and 161.9 eV due to reduced (S^2−^) and pristine sulfur, respectively. Single and broad Si2p peak at 101.7 eV could be assigned to Si-C, Si-C/Si-N, Si-N and Si-O/Mo-Si peaks at 100.2, 101.4, 101.7 and 102.1 eV, respectively^57^. The broad oxygen peak at 531 eV could be fitted by doublets Mo-O_2_ (530.2 eV and 531 eV) and more electronegative Mo-O_3_ (at 531.6 eV). High-resolution C1s peak could be deconvoluted into Mo-C, Si-C and -sp^2^ carbon at 282.7, 283.4 and 284.4 eV, respectively. Therefore, a possibility of chemical interaction/bond formation exists between MoS_2_ sheets and SiCN ceramic, confirmed by the presence of Mo-C, Mo-Si bonds.

**Figure 3:**
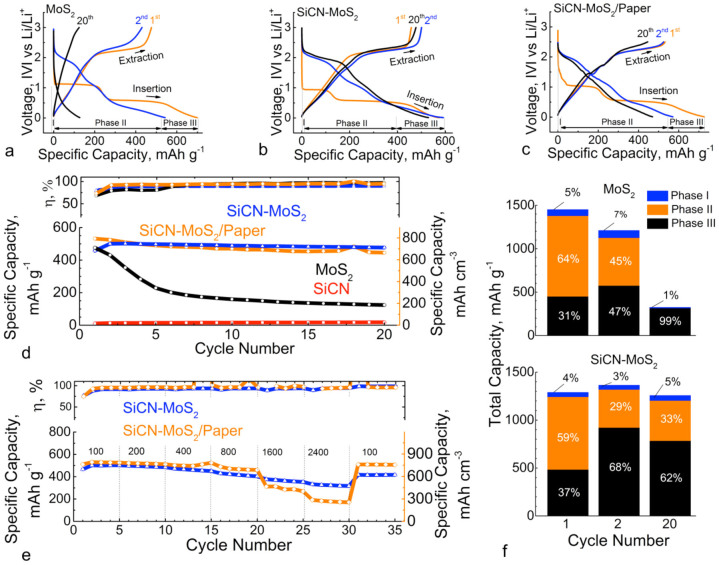
Electrochemical data. Comparison of first, second and twentieth electrochemical cycle (voltage-capacity plots) of half-cells of (a) acid-treated MoS_2_ (85 mA.g^−1^), (b) SiCN-MoS_2_ (at 116 mA.g^−1^) composite and (c) SiCN-MoS_2_ freestanding composite paper electrodes. (d) Electrochemical cycling performance of traditional electrodes (acid-treated MoS_2_, SiCN, SiCN-MoS_2_ composite) and freestanding electrode (SiCN-MoS_2_ paper) for initial 20 cycles. (e) C-rate cycling performance comparison of SiCN-MoS_2_ composite and SiCN-MoS_2_ paper. (f) Comparison of total capacity for initial (1^st^ and 2^nd^) and final (20^th^) cycles and contribution of various reactions for acid-treated MoS_2_ and SiCN-MoS_2_ electrodes.

Electrochemical performance of acid-treated MoS_2_ and SiCN-MoS_2_ composite electrodes was studied and compared by performing galvanostatic cycling experiments of the half-cell under constant current conditions. When cycled between 10 mV to 3 V at 100 mA.g^−1^ constant current, first cycle discharge capacity of 698.9 mAh·g^−1^ (or 89.84 mAh·g^−1^_electrode_, i.e., when normalized with respect to total weight of the electrode including the current collector) and charge capacity of 476.3 mAh·g^−1^ (or 61.2 mAh·g^−1^_electrode_) were observed for the acid-treated MoS_2_ specimen (Figure 3). Cycle hysteresis of approximately 0.5V was also observed, which is typical of a conversion type reaction. After 20 cycles, the reversible capacity decayed to 124.2 mAh·g^−1^ or 26% of initial capacity, which was slightly lower than the theoretical capacity of 167 mAh·g^−1^ for bulk MoS_2_ (considering one mole of Li^+^ intercalation reaction). Under cycling at similar conditions, the SiCN-MoS_2_ traditional electrode exhibited first cycle discharge capacity of 574.1 mAh·g^−1^ (or 126.1 mAh·g^−1^_electrode_) and reversible capacity of 457.6 mAh·g^−1^ (or 100.5 mAh·g^−1^_electrode_). During the cycling test for 20 cycles the initial reversible capacity was retained at 477 mAh·g^−1^ (or 104.7 mAh·g^−1^_electrode_). For the freestanding SiCN-MoS_2_ composite paper electrode cycled under similar conditions, the first-cycle discharge capacity of 726.4 mAh·g^−1^ and reversible capacity 532.1 mAh·g^−1^ were observed. These capacities were retained after 20 cycles at 445.6 mAh·g^−1^ (73% of initial value). Anticipated intercalation and conversion reactions for exfoliated MoS_2_ are given as:

At counter electrode,
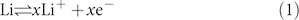
Intercalation at working electrode
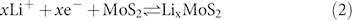
Conversion reaction at working electrode,
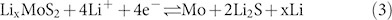
Where, *x* represents the number of moles of intercalating Li-ions (or corresponding electrons) in the host material. Based on varying slope of capacity versus voltage plot, the cycling profile for these electrode materials was divided into three phases, as shown Figures 3(a) and 3(c).^50^,^58^,^59^,^60^ Individual contribution of each phase to overall capacity of initial and last tested cycles for MoS_2_ and SiCN-MoS_2_ are shown in Figure 3(f).

The changing voltage slope and plateaus observed in the cycled electrodes can be studied further by differentiating capacity with respect to the voltage as shown in Supplementary Figure S5. The plateaus or steep slope observed in the cycling plot, appear as distinct peaks (labeled) in corresponding dQ/dV plots. Varying intensity of each peak indicates changing rates of Li-ion interaction within the host material, further signifying various reactions and structural phases involved. First-cycle dQ/dV for exfoliated MoS_2_ showed prominent lithiation peaks at approximately 1.1 V and 0.57 V, corresponding to conversion reaction whereby Li*_x_*MoS_2_ converts to Mo metal and Li_2_S (Equation 3). The delithiation peak at 2.2 V is known to originate from the crystalline nature of MoS_2_.^12^ For the second cycle, prominent lithiation peaks were observed at approximately 1.9 and 0.3 V, and an delithiation peak was observed at 2.3 V. These results are consistent with data from literature on transition metal sulfide electrodes.^9^,^12^,^17^ For the first-cycle of SiCN-MoS_2_ electrode, a slight shift in potential of lithiation peaks were observed at approximately 0.93 V, 0.56 V, 0.3 V, and 40 mV (when compared to MoS_2_ first-cycle behavior). For the second cycle, major reduction peaks were observed at approximately 2 V, 1.1 V, and 0.4 V, but no change was observed in delithiation peak position. The SiCN-MoS_2_ freestanding paper electrode experienced reduction and oxidation peaks similar to SiCN-MoS_2_ traditional electrodes. Lithiation (0.05 and 0.75 V) and delithiation (0.15 V) peaks of rGO (graphene)^61^ were not distinguishable from primary peaks of SiCN-MoS_2_ because contribution of rGO (10% or less mass loading) to overall specific capacity was negligible.

Figure 3 (d) compares the charge capacity of SiCN-MoS_2_ composite (traditional and paper electrodes) to acid-treated MoS_2_ and ‘neat’ SiCN ceramic electrodes cycled under similar conditions. The figure shows that SiCN-MoS_2_ electrodes had approximately 26% higher capacity retention at 20 cycles when compared to MoS_2_. In addition, the ‘neat’ SiCN electrode had negligible reversible capacity of approximately 20 mAh·g^−1^. Figure 3 (e) shows the C-rate performance of SiCN-MoS_2_ traditional and paper electrodes. First-cycle charge capacities were 465.9 mAh·g^−1^ and 530 mAh·g^−1^ for traditional and paper electrodes, respectively. When current density increased to 2400 mA.g^−1^, respective charge capacities dropped to 326.4 mAh·g^−1^ and 191.9 mAh·g^−1^. However, most of the capacity was recovered when the current density was decreased to 100 mA.g^−1^, reaching 414.8 mAh·g^−1^ (83% retained) and 509.2 mAh·g^−1^ (96% retained) for traditional and paper electrodes, respectively. A summary of the electrochemical data is presented in Table 1.

**Figure 4:**
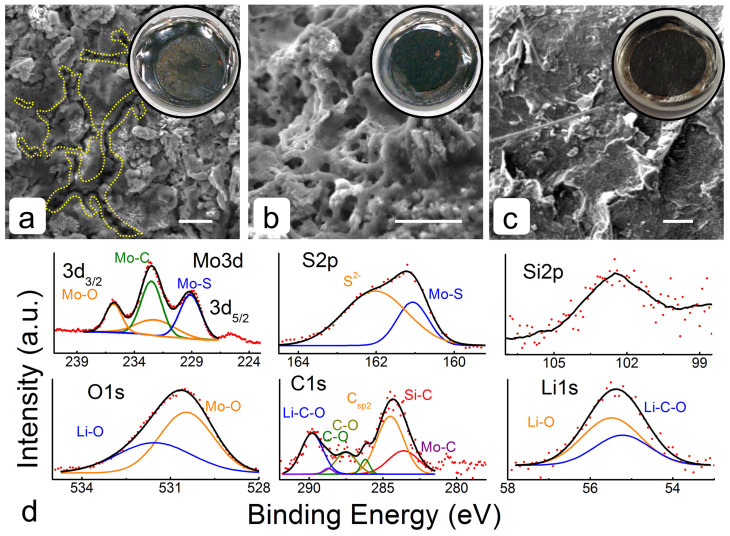
Post electrochemical analysis. SEM images of disassembled cells (a) acid-treated MoS_2_, (b) SiCN-MoS_2_ composite and (c) SiCN-MoS_2_ paper electrodes after testing for 20 cycles. The insert shows digital images of corresponding electrode surfaces. (d) Elemental X-ray photoelectron spectra of the dissembled SiCN-MoS_2_ composite electrode show Mo, S, and C phase modifications with additional Li peaks. Scale bar is 10 *μ*m.

After 20 electrochemical cycles, the half-cells were disassembled and the electrode was recovered (delithiated state) for further investigation of structure and chemical composition. Figures 4(a through c) are SEM images of the disassembled cells. The inserts are corresponding optical camera images in which the MoS_2_ electrode exhibited signs of microcracks, coating delamination and slight discoloration, suggesting susceptibility to volume and chemical changes during the intercalation (Phase I) and conversion (Phase II) reactions. However, SiCN-MoS_2_ cycled electrodes appeared to be largely intact without any change in color from the original (pre-cycled) state. SEM imaging also provided additional details regarding the effects of electrochemical cycling at the micro-scale. The cycled MoS_2_ electrode showed uneven surface and mud cracks (Figure 4(a)), while the SiCN-MoS_2_ electrodes (traditional and paper) were relatively more porous and consisted of an uninterrupted solid electrolyte interphase (SEI) film on the active material (Figures 4 (b,c)).

The chemical composition of the cycled SiCN-MoS_2_ electrode was also analyzed by XPS. As shown in Figure 4(d) the Mo3d elemental peak evolved into three slightly overlapping peaks at 229.13 eV, 232.48 eV and 235.81 eV. Low energy peaks at 229.13 eV and 232.1 eV were attributed to Mo-S and Mo-O type bonds, respectively. Higher energy doublets at 232.48 eV and 235.81 eV could be assigned to the more electronegative Mo-O_2_/MoO_3 _and Mo-O_x_ species, respectively.^54^,^55^,^56^,^57^ Both sulfur peaks shifted to higher energies of 161.06 eV and 162.02 eV, likely due to S^2−^ and polysulfide (Li-S) entities, respectively.^56^,^57^ Broad silicon elemental peak with weak intensity could be attributed to Si-C and Si-O type bonds. The shifted oxygen peak at 530.5 eV could be fitted by a peak at 530.46 eV due to Mo-O type bonds and fitted by a peak at 531.54 eV due to multiple entities of more electronegative Li_3_PO_4_, Li_2_CO_3_, Li_2_O, LiOH, and Mo-O_3 _compounds.^56^ High-resolution C1s peak was deconvoluted into Mo-C, Si-C, C-sp^2^, C-O, and Li-C-O at 282.7 eV, 283.6 eV, 285.5 eV, 286.2 eV, 287.5 eV, and 289.8 eV, respectively. Lastly, the Li1s elemental peak at 55.4 eV was assigned to Li-C (Li_2_CO_3_) and Li-O (Li_2_O) at 55.22 eV, and 55.48 eV, respectively.

### Conclusion

Acid-treated MoS_2_ was utilized to prepare polysilazane/MoS_2_ composite, which upon pyrolysis in an inert environment, resulted in formation of SiCN-MoS_2_ nanosheets. Electron microscopy revealed uniform distribution of SiCN-MoS_2_ stacked sheets in the composite. XPS analysis revealed formation of Mo-C and Mo-O bonds, indicating chemical bonding of the SiCN’s carbon phase with molybdenum atoms. Electrochemical performance of the composite was studied as working electrode in LIB half-cell, revealing an increasingly stable cycling and higher capacity retention compared to ‘neat’ MoS_2_ after 20 cycles. The contribution of electrolyte decomposition (Phase III) to overall capacity decreased for SiCN-MoS_2_ electrodes. This decrease is one of the reasons for decreased first-cycle loss and increased capacity retention for SiCN-MoS_2_ composite. Additionally, the electrically conductive nature of SiCN ceramics may have resulted in faster Li-ion diffusion. These observations suggests that chemical interfacing of MoS_2_ surfaces with precursor-derived ceramics is a promising approach toward achieving stable Li-cycling in rechargeable batteries composed of transition metal sulfide electrodes.

## Author Contributions

**Author contribution** R.B. prepared SiCN-MoS_2_ composite, traditional electrodes, performed SEM and XPS analysis. L.D. prepared paper-based electrodes and their XRD, TGA and TEM analysis. U.B. assisted R.B. and L.D. with coin cell preparation and electrochemical testing. G.S. conceived the idea, designed the experiments and wrote the manuscript with inputs from R.B. and L.D. All authors discussed the results and commented or revised the manuscript.

## Supplementary Material

Supplementary Information Supplementary Information

## Figures and Tables

**Table 1:** Summary of the electrochemical data and comparison with literature on other exfoliated-MoS_2_ based electrodes

Electrode Type	Active Material (mg)	First Reversible Capacity (mAh·g^−1^)	ICL (%)	First Reversible Capacity (mAh·g^−1^_electrode_)	Reversible capacity in (mAh·g^−1^_electrode_) (20 cycles)
MoS_2_ traditional	1.66	595.3	32	61.22	16
SiCN traditional	1.12	13	86.6	7.17	2
SiCN-MoS_2_ traditional	3.49	572.05	20.2	100.5	104.8
SiCN-MoS_2 _paper electrode	6.4	623.5	27	498.8	417.8
MoS_2_ exfoliated traditional (Ref. 16)	N.A.	~766	32	N.A.	N.A.
MoS_2_ bulk traditional (Ref. 16)	N.A.	275	54	N.A.	N.A.
MoS_2_ paper (Ref. 20)	N.A.	375	N.A.	~375	~125
MoS_2_/CNT (Ref. 20)	N.A.	280	N.A.	~280	~225
