# Supplementary material for: Polymer-Derived Ceramic Functionalized MoS2 Composite Paper as a Stable Lithium-Ion Battery Electrode
Source: Sci Rep. 2015 Apr 8;5:9792. doi: 10.1038/srep09792 (PMC4389211; doi:10.1038/srep09792)
Supplement: Supplementary Information [file srep09792-s1.pdf]

SUPPLEMENTARY INFORMATION

Polymer-derived Ceramic Functionalized MoS_2 Composite Paper as a Stable Lithium-ion Battery Electrode

R. Bhandavat[¥], L. David[¥], U. Barrera and G. Singh*

Department of Mechanical and Nuclear Engineering, Kansas State University, Manhattan, KS 66506, USA

Tel.: +1-785-532-7085

Fax: +1-785-532-7057

*E-mail: gurpreet@ksu.edu

[¥] *Both authors contributed equally*

I. THERMOGRAVIMETRIC ANALYSIS

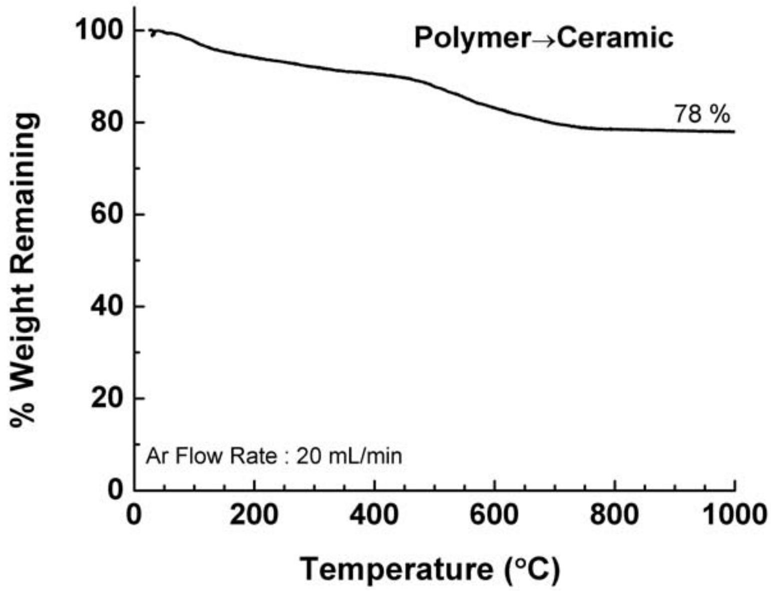

SUPPLEMENTARY FIGURE S1: TGA data performed in flowing Ar gas for cross-linked polysilazane, shows polymer to ceramic transformation. The polymer to ceramic yield was approx. 78 %.

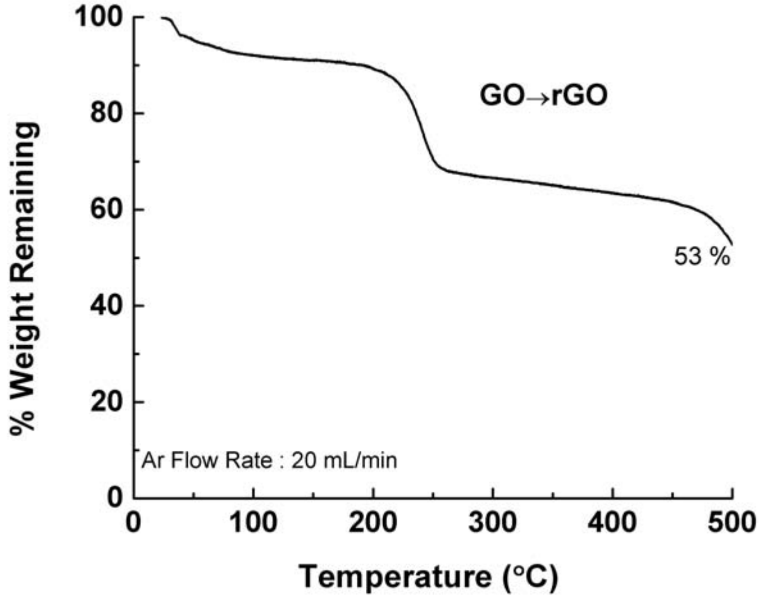

SUPPLEMENTARY FIGURE S2: TGA data performed in flowing Ar gas, shows thermal reduction of graphene oxide (GO) to reduced-graphene oxide (rGO). The yield was approx. 50 % when thermal reduction is performed at 500 °C.

II. X-RAY DIFFRACTION DATA

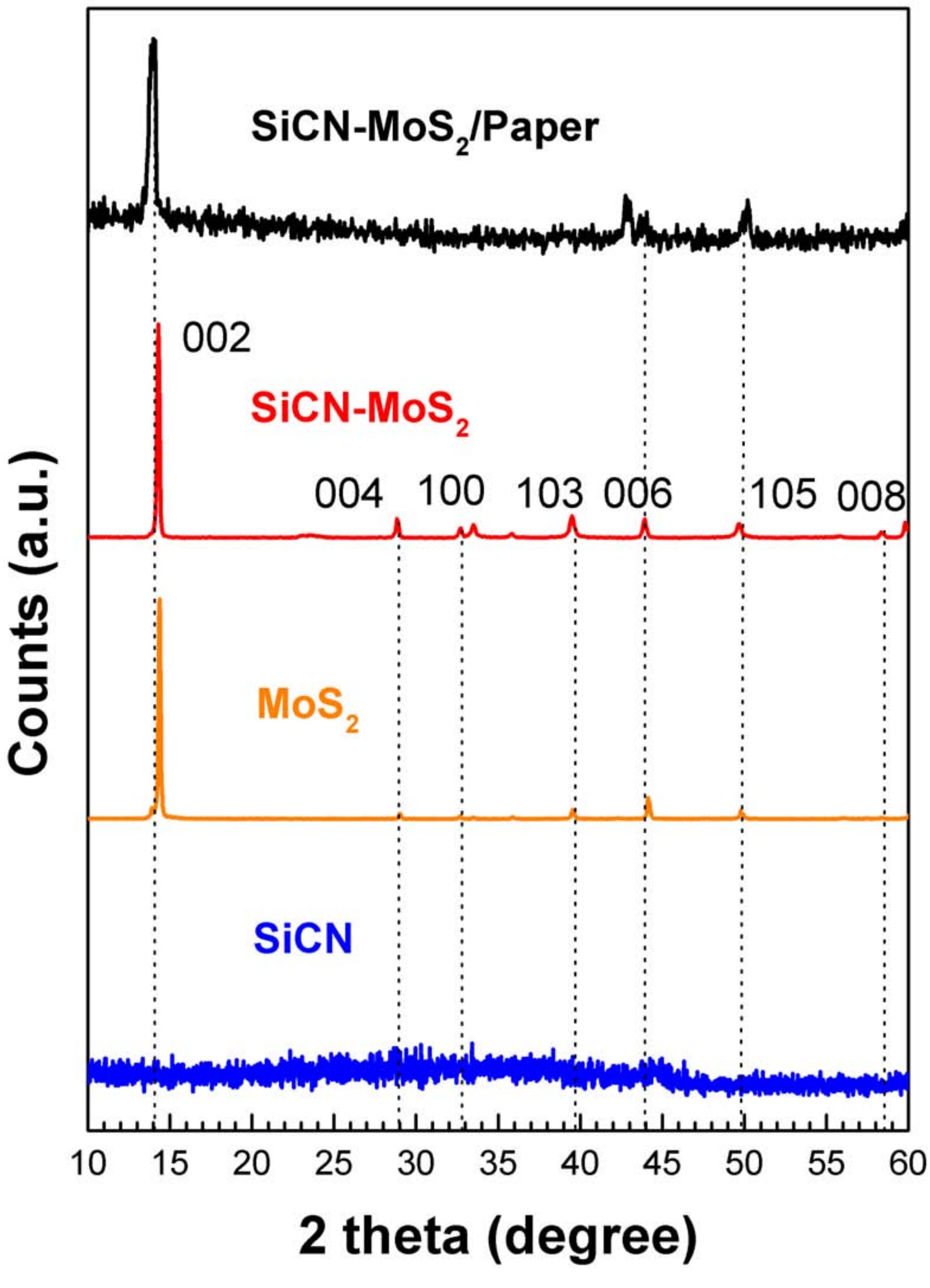

SUPPLEMENTARY FIGURE S3: XRD diffraction data comparison for various electrode specimens prepared in this study. Polymer-derived SiCN ceramic is amorphous while crystalline peaks for MoS_2 could be clearly visualized.

III. X-RAY PHOTOELECTRON SPECTROSCOPY

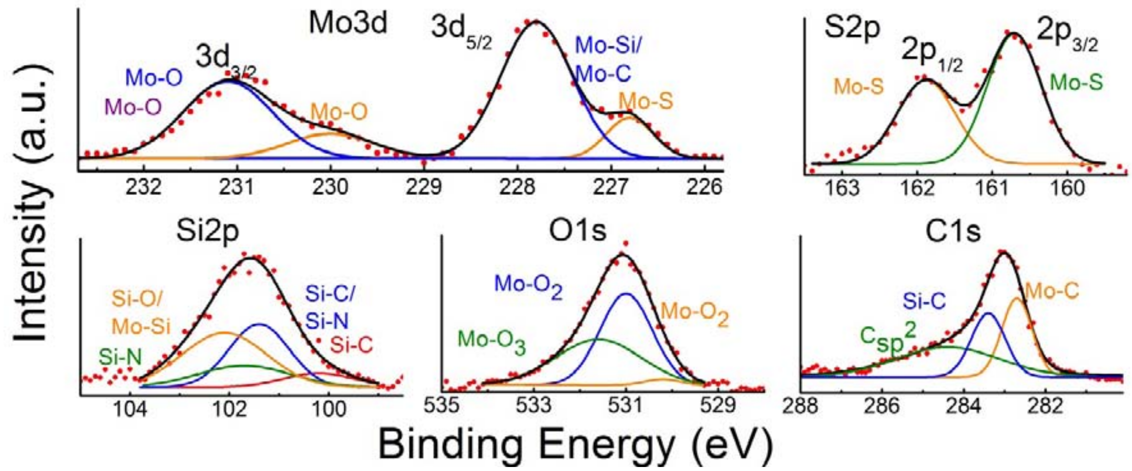

SUPPLEMENTARY FIGURE S4: High-resolution X-ray photoelectron spectra of SiCN-MoS_2 composite nanosheets showing the characteristic MoS_2 and additional peaks that emerge as a result of chemical functionalization with SiCN ceramic.

IV. ELECTROCHEMICAL DIFFERENTIAL CAPACITY CURVES

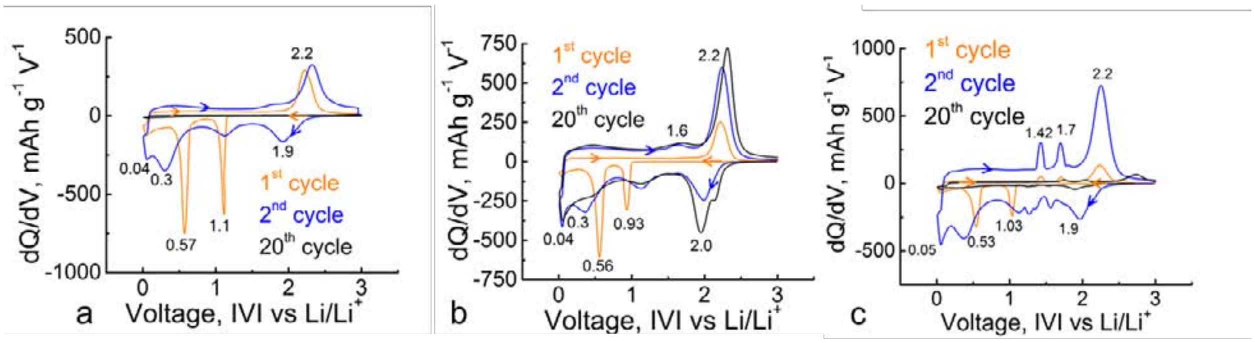

SUPPLEMENTARY FIGURE S5: Differentiated capacity with respect to corresponding operating voltage (dQ/dV) for (a) acid-treated MoS_2, (b) SiCN-MoS_2 composite and (c) SiCN-MoS_2 free-standing composite paper.
